# On Redefining the Body Image Satisfaction Questionnaire: A Preliminary Test of Multidimensionality

**DOI:** 10.3390/healthcare9070876

**Published:** 2021-07-13

**Authors:** Filipe Rodrigues, Diogo Monteiro, Pedro Flores, Pedro Forte

**Affiliations:** 1 Sport Science School of Rio Maior (ESDRM-IPSantarém), 2040-413 Rio Maior, Portugal; frodrigues@esdrm.ipsantarem.pt; 2 Life Quality Research Center (CIEQV), 2040-413 Santarém, Portugal; 3 ESECS, Polytechnique of Leiria, 2411-901 Leiria, Portugal; diogo.monteiro@ipleiria.pt; 4 Research Centre in Sports, Health and Human Development (CIDESD), 5001-801 Vila Real, Portugal; 5 Department of Sports, Douro Higher Institute of Educational Sciences, 4560-708 Penafiel, Portugal; pedro.flores@iscedouro.pt; 6 Departamento de Desporto e Educação Física, Instituto Politécnico de Bragança, 5300-253 Bragança, Portugal

**Keywords:** self-image, psychometric analysis, exploratory analysis, bifactor modeling

## Abstract

The aim of the present study was to examine the Body Image Satisfaction Questionnaire (BISQ) as a multidimensional instrument, designed to measure individuals’ body image satisfaction. A sample of 790 Portuguese healthy adults (female = 399; male = 391) aged 18 and 49 years old (*M* = 28.61, *SD* = 7.97) completed the BISQ. Exploratory factor analysis of the BISQ provided initial psychometric validity for a five-factor model assessing five dimensions of body image, namely, face, upper torso, lower torso, lower body, and overall body appearance. Confirmatory factor analysis supported this five-correlated model, in which a bifactor model provided the best fit to the data, defining a body image satisfaction factor and five specific factors. The BISQ clearly distinguished between various dimensions of body image satisfaction and showed satisfactory psychometric quality through factor analyses. This measure may have a broad application for research and practice, as a tool for capturing individual body image satisfaction.

## 1. Introduction

Body image (BI) is a self-perception about a person’s physical appearance [1] that has been heavily influenced by (and in) society through social media, television, interpersonal connections, and covers in magazines and printed media [2]. BI can be affected by cultural background and social pressures to present oneself as similar to famous personalities [3]. Additionally, BI is closely related to self-esteem, psychological health, and eating disorders [4,5].

According to Eggermont [6], BI is defined as a positive or negative satisfaction with one’s own body size and shape. Moreover, BI is a multidimensional concept, in which cognitive (e.g., self-esteem, self-image, attitudes), affective (e.g., positive and negative affect, mood), and behavioral (e.g., eating disorder, food craving) factors are examined to understand people with healthy or unhealthy perceptions of their bodies. That said, the BI is a dynamic concept that alters itself along the life cycle according to internal and external sources [7]. At a personal level, an individual’s experience of a positive or negative body image has some association with different components of behavior. For example, previous cross-sectional studies have found positive associations between body dissatisfaction and both eating disorders in 586 outpatients with eating disorders [8], and body mass index (BMI) in boys and girls aged 9–18 years from the Growing Up Today Study, a national prospective cohort of U.S. youth (*n* = 16,882) [9]. Other cross-sectional studies have found a positive correlation between body image satisfaction and healthy eating habits and self-esteem in girls and boys transitioning from early to mid-adolescence [10]. Thus, the assessment of body image is important, as it can contribute to an understanding of how different body parts may be related to different behavioral, cognitive, and perceptual determinants, such as healthy or unhealthy eating, positive or negative affect, and positive or negative self-esteem.

Recently, Kling et al. [11] conducted a systematic review of validated measures of body image. Among them, these authors pointed out eight measures (i.e., the Body Appreciation Scale, the Body Esteem Scale for Adolescents and Adults, the Body Shape Questionnaire, the Centre for Appearance Research Valence Scale, the Drive for Muscularity Scale, two subscales of the Eating Disorders Examination Questionnaire, one subscale of the Eating Disorder Inventory 3, and two subscales of the Multidimensional Body Relations Questionnaire) with evidence of reliability and validity. These authors in their systematic review concluded that nearly all valid self-reported measures focused on BI dissatisfaction rather than satisfaction and that “Future studies should primarily focus on extending the available evidence for already existing measures rather than developing new measures of evaluative body image” (page 182). Considering that dissatisfaction (i.e., “I am unattractive”) is not the opposite of satisfaction (i.e., “I am satisfied with by body shape”), measures of BI satisfaction should be psychometrically examined and validated. According to our search, the Body Image Satisfaction Questionnaire (BISQ; [12]), while used in recent studies (e.g., [13]) has not undergone this psychometric evaluation.

The BISQ is a 23-item measure aiming to rate participants’ satisfaction towards different body parts (e.g., nose, hair, shoulders) as well as their overall body image satisfaction (e.g., vitality, body shape, looks). Prior studies found measures of BI satisfaction to be valid in examining its association with behavioral aspects, such as anorexia, body dysmorphia, in which lower scores of BI satisfaction could be related to behaviors that negatively impact physical and mental health [14]. However, to the best of our knowledge, this measure has never been subjected to factor the analytic method. In fact, a deeper examination of this measure and the studies that have used it empirically seem to consider this 23-item scale as unidimensional, when there are conceptual differences among the items (e.g., nose, body shape, vitality). More specifically, previous studies (e.g., [13]) seem to use this measure as an overall perception of body image satisfaction, when in fact, the measure taps not only perceptions of different body parts, but also the experience of vitality, and overall appearance satisfaction. This unidimensional approach seems to limit the interpretation of body image, especially as it has been described by several other authors (e.g., [14]) as a multifaceted construct. Falling short to distinguish the various dimensions of body image could hinder the essential role body image plays in the general population’s health and well-being.

These observations raise essential questions related to: (a) whether perceptions of body image are better represented by one (i.e., overall body image satisfaction) or several (e.g., body appearance, facial appearance, specific body segment satisfaction) dimensions; (b) whether these dimensions co-exist as a global entity, including specificities that are mapped by specific dimensions of body image satisfaction. We designed the current study specifically to address these questions, while focusing on respondents on the Body Image Satisfaction Questionnaire (BISQ; [12]), designed to assess self-perception of both specific body parts and satisfaction with overall body appearance, physical fitness, and vitality.

Considering that the BISQ inquires of respondents what their satisfaction perceptions may be regarding both separate body parts and general appearance satisfaction, it is hypothesized that factor analysis would reveal several factors or dimensions of body image satisfaction, rather than just one. Specifically, since some items are specific to face (i.e., nose, hair, ears), and others to lower body (i.e., glutes, thighs, legs), we expected a multi-factor model to emerge—one that measures different body part dimensions.

## 2. Materials and Methods

### 2.1. Participants

To examine the reliability and validity of the BISQ, a non-experimental, cross-sectional, and correlational study design was followed. Data from 790 individuals (female = 399; male = 391) aged between 18 and 49 years (*M* = 28.61, *SD* = 7.97) was used to statistically analyze the instrument’s validity and reliability. To correctly perform the study, Portuguese individuals were invited to participate voluntarily, according to the following inclusion criteria: all participants were at least 18 years of age and completed the questionnaire voluntarily. To provide generalizable results, female and male participants were included. Excluding criteria were underage individuals, any participant that did not declare informed consent and that did not answer the complete questionnaire (missing values above 5%). Data were collected between January 2018 and March 2020 (before COVID-19 restrictions).

### 2.2. Procedures

Data collection procedures were conducted in accordance with the Declaration of Helsinki and its later amendments. Additionally, approval of the Institutional Research Ethics Committee was obtained prior to data collection from the Douro Higher Institute of Educational Sciences. Data were collected by convenience in the North region of Portugal at schools, health centers, and other public organizations to gather a relatively large and diversified community sample of participants, to maximize the generalizability of our results. We contacted gym and health club centers, higher education institutions, and public working organization by convenience to collect data. The study’s objectives and data collection procedures were explained to directors and managers of these institutions and organizations. After approval, directors and managers contacted their employees during different periods of the day and were asked to participate voluntarily in this study, reinforcing anonymity and confidentiality. Potential participants were first informed about the main objective and the topics of the study. Individuals who agreed to participate had to check a box within the online survey, before moving on to completing the questionnaire.

### 2.3. Measures

The BISQ [12] Portuguese version [13] was used to rate how individuals experience their satisfaction with each body part. Participants were asked to rate “For each item, cross the number that corresponds to the type of satisfaction you feel related to your body”. The 23 items were related to facial parts (item examples: teeth, hair, eyes, nose), body parts (item examples: glutes, arms, chest), and overall appearance (item examples: physical fitness, height, vitality, body shape). Participants were asked to rate the extent to which they were satisfied with their body parts on a five-point scale ranging from 1 (“I don’t like and would like to be different”) to 5 (“I consider myself favored”).

The original scale encompasses two additional items using the stem “I feel…” to assess how participants feel related to their height (i.e., Tall, Short, or I have the ideal height) and weight (i.e., Fat, Slim, I have the ideal weight). These items were not considered for modelling since they were nominal variables and had a 3-point scale range compared to the 5-point range in the previous 23 items. Additionally, these items overlap since height and weight are already considered in the 23-items measure.

### 2.4. Statistical Analyses

Exploratory and confirmatory analyses were conducted in Mplus 7.3 [15] using the Robust Maximum Likelihood (MLR) estimator as it provides tests of model fit and standard errors that are robust to the non-normality of the data. Full Information robust Maximum Likelihood (FIML) was used to handle the small amount of missing data at the item level (missing at random = 3%) as proposed by several authors [16,17].

The examination of the BISQ was conducted in two phases. In the first one, several exploratory models were tested and compared. The best fitting model was retained for the confirmatory factor analysis. Then, after examining the measurement model, and providing acceptable fit, second order and bifactor model specifications were tested to examine dimensionality of the BISQ. Following previous applications of CFA specifications [18,19], all models were specified with target rotation procedures [20]. In the second order model, all factors were regressed into a single factor, representing and overall satisfaction of body image. In the bifactor CFA model, items were loaded onto their predefined specific factors and onto a global factor, and all specific factors could correlate freely. In the second phase, to investigate factor structure of the retained model specification, the best CFA model of the BISQ was examined for convergent and discriminant validity, internal consistency, and factor loadings and uniqueness.

With respect to the exploratory factor analysis evaluation, several indices were considered. First, the criteria of Eigenvalues >1 and visual inspection of the screen plots were used to determine the appropriate number of factors [21]. Second, geomin loadings were examined considering the level of significance (*p* < 0.001) and cross-loadings. Cross-loadings below 0.15 should not be considered as problematic, retaining the item in the factor where it loaded the highest [21].

Measurement model evaluation was carried out following the traditional and incremental indexes: Comparative Fit Index (CFI), Tucker Lewis Index (TLI), Standardized Root Mean Square Residual (SRMR), Root Mean Square Error of Approximation (RMSEA), and its respective 90% Confidence Interval (CI90%). The qui-squared test (*χ*^2^) and the degrees of freedom will be reported for visualization purposes, as they are both affected by the complexity of the model and sample size [21]. Considering traditional cutoffs reported elsewhere [21,22,23], CFI and TLI were considered acceptable and adequate with values over or equal 0.90, while values below or equal 0.08 were indicative of good fit for SRMR and RMSEA.

Based on previous literature [19,24], the selection of the most optimal model should not be solely based on the traditional and incremental fit indexes, but should be coupled with the examination of other relevant theoretical and statistical measures and key estimates (i.e., factor loadings, factor correlations). Hence, the correlation matrix of the best fitting CFA model was examined. Looking at item loadings, the pattern matrix was inspected and items loading >0.50 were retained as they explained at least 25% of variance in the latent factor [21]. Cross-loading items were accepted if differences in loadings were below 0.15, retaining in the pre-specified factor. The internal consistency (i.e., reliability) of items assigned to a factor was assessed using Raykov formula, considering 0.70 as a minimum acceptable level [25]. In the bifactor model, omega coefficients were calculated since when multidimensional data are fit to a bifactor model, it is essential for scholars to examine the strength of the resulting general and group factors, as its value is influenced by all modeled sources of common variance [26].

## 3. Results

The exploratory factor analysis showed that the five-factor model would provide the best fit to the data. Thus, analyses moved forward with the five-factor solution, as it displayed five possible dimensions with eigenvalues above one and goodness-of-fit indices showed acceptable fit (Table 1).

The factor loadings from the five-factor EFA solution were examined for possible cross-loadings and non-significant loadings. Looking at the results displayed in Table 2, the first seven items loaded significantly F1, while item 8–10 loaded significantly F2. Item 11–13 as well as item 17 were positive and significant in F3, whereas item 14–16 loaded significantly F5. The remaining items (i.e., 18–23) loaded significantly F4. Some cross loadings were found (e.g., Item 1, Item 6); however, since differences between factor loadings were below 0.15, items were retained in the factor where they loaded the most as proposed in the literature. Items displayed means above midpoint (range 3.00–4.05) and showed normal distribution since skewness and kurtosis were contained within cutoffs. Considering these results, a five-correlated CFA model was tested to confirm our hypothesis (Table 2).

The five-correlated CFA model provided acceptable fit, in which TLI was very close to achieving the minimum score for adequate (0.899). Nonetheless, CFI, SRMR, and RMSEA were contained within cutoffs, suggesting that the model fit the data well (see Table 1). Factor loadings (see Table 3) were above 0.50, explaining at least 25% of variance in the latent factor. Additionally, all items loaded significantly (*p* < 0.001) the predefined factor, showing suitability of the item distribution according to the EFA results. Last, internal consistency was achieved since composite reliability coefficients were above 0.70, ranging from 0.77 to 0.90 as seen in Table 3. Considering these results, analyses move forward on testing a second order and a bifactor CFA model.

The second-order model did not provide acceptable fit as seen in Table 1 (model 8). Specifically, it did not show good goodness-of-fit indexes, since CFI and TLI were below cutoffs, and SRMR and RMSEA were above previous reported guidelines. Thus, no further analysis was conducted considering this model.

With respect to the bifactor model specification, results showed acceptable fit to the data (see Table 1, model 9). In fact, it provided a better fit compared to the five-correlated CFA model. The bifactor-CFA model is of great theoretical importance since it provides a direct estimate of the global body image satisfaction dimension and the hypothesized specific factors. Standardized parameter estimates for this final model, are reported in Table 3. The global factor was relatively well-defined (|λ| = 0.35 to 0.83, *M* = 0.58, ω = 0.96) with the positive and significant contribution by all scale items. Apart from the global factor, most specific factors retained an acceptable degree of specificity (face: |λ| = 0.21 to 0.68, *M* = 0.44, ω = 0.83 upper torso: |λ| = 0.56 to 0.68, *M* = 0.64, ω = 0.94; lower torso: |λ| = 0.45 to 0.65, *M* = 0.52, ω = 0.90; lower body: |λ| = 0.56 to 0.73, *M* = 0.64, ω = 0.90; body appearance: |λ| = 0.29 to 0.65, *M* = 0.52, ω = 0.93), suggesting that there is meaningful specificity once the variance explained by the general factor is accounted for. Overall, based on better model fit and theoretical representation, the bifactor-CFA solution seems to represent well the dimensionality of each factor, as well as the global experience of body image satisfaction.

In the last step, descriptive statistics and correlations were calculated. Again, dimensions of body image satisfaction were above scale midpoint, ranging from 0.328 to 3.75. All factors displayed normal distribution since skewness and kurtosis were contained within cutoffs. The correlations were all positive and significant at 0.001, as seen in Table 4, specifically: (a) face was positively and significantly correlated with upper and lower torso, lower body, and body appearance; (b) upper torso was positively and significantly associated with lower torso, lower body, and body appearance; (c) lower torso was positively and significantly correlated with lower body and body appearance; and, (d) lower body was positively and significantly associated with body appearance. The highest correlation was between lower body and lower torso (0.77, *p* < 0.001).

## 4. Discussion

The main objective of the current investigation was to redefine and validate an instrument to measure BI satisfaction, which is named the BISQ. Exploratory factor analysis, CFA, and bifactor CFA revealed that a five-factor solution assessing different dimensions of body image satisfaction (i.e., face, upper torso, lower torso, lower body, body appearance) produced the best fit. Hence, the current results support the earlier hypothesis and advanced theory on the assessment of body image satisfaction based on the BISQ, an under-researched measure. These factors were all positively and significantly correlated at a moderate to high level, suggesting the existence of BI satisfaction between factors.

Using exploratory factor analysis on the 23-item matrix related to the BI satisfaction, we have examined a structure-validated self-report, the BISQ [12] in a sample of healthy Portuguese adults. The disclosure of five factors namely, face, upper torso, lower torso, lower body, and body appearance has confirmed our hypothesis regarding the multidimensional structure of the BISQ, which confirms multidimensionality proposed by Flores et al. [13]. Being distinct from each other, the five-factor model showed a satisfactory fit, which is consistent with the proposition that the BISQ measures different parts that are related to how individuals perceive their own embodiment, especially, but not exclusively, their BI [14]. In addition, a bifactor CFA model provided a better fit to the data than did the five-correlated factor model. Note that while the five-correlated CFA factor model provided acceptable fit and was consistent with our hypothesis, the bifactor (i.e., one general factor, and five specific factors) provided better fit meeting all pre-specified criteria therefore offering a more parsimonious model [21]. Hence, current results support the existence of five specific factors and a global factor related to the overall perception of body image satisfaction. In a broader and theoretical sense, BI can be seen at the specific level of body appearance satisfaction (e.g., face), but also include experiences related to one’s physical functional satisfaction (e.g., body image per se). Thus, our data suggest that participants were able to differentiate their BI satisfaction at the general level, but additionally were capable of defining distinct factors related to body appearance. The definition of the factors is based on the items measuring different body parts and are not defined nor conceptualized as strict constructs. Hence, we should mention that this is not a theoretical study as to precisely defining each factor, but rather an exploratory study, considering the hypothesis of a multidimensional measure. Thus, we recommend more studies on the BISQ to determine the best fitting conceptualization for each factor.

As stated by Avalos et al. [27] in order to contribute to the development of BI research, it is imperative that measures of positive body image are created and evaluated. Therefore, our study validated a measure of body satisfaction (i.e., the BISQ) that contains five central aspects of BI satisfaction and examined its psychometric properties for the first time. Generally, current results demonstrated that the BISQ has good psychometric support among the Portuguese adult population, as its hypothesized factor structure was supported, factor loadings were internally consistent across the correlated five-factor structure, and it demonstrated evidence of construct validity. Our results also revealed that some BISQ items provided a better reflection of individuals’ global body image satisfaction than of their specific assessment of each dimension. For instance, item 21 (physical fitness), item 9 (chest), or item 7 (facial aspect), might provide a stronger measure of global body image satisfaction for this sample of individuals whom daily work involves relationships with others. Clearly, the present study needs to be replicated using other samples to test this interpretation. Nonetheless, all items significantly loaded the global factor, as well as the specific factor, and factors displayed acceptable composite reliability coefficients. Hence, while this study is a preliminary analysis of the BISQ as a multidimensional measure, current results shed new insights on how this measure can assess different dimensions of body image satisfaction.

The BISQ was developed as a measure of the body image satisfaction [12]. As such, the present findings are somewhat new compared to the original work, as we tested a multidimensional model rather than considering body image satisfaction as a composite score, it has been used in previous studies [12]. The strong factor loadings found in the general factor and specific factors may indicate that face, upper and lower torso, lower body, and body appearance share some underlying variance (e.g., overall body appearance). Notably, this may enable scholars to test whether other factors can contribute to the prediction of meaningful outcomes over and above these factors (e.g., Body Mass Index). Moreover, a bifactor structure augments the BISQ model incorporating recommendations proposed by Marsh et al. [28] regarding the use of higher-order multidimensional constructs. For example, this bifactor structure retains the conceptual uniqueness and original work of [12], whilst providing an empirically testable model, moving forward, with greater parsimony and bandwidth in the general population with respect to the measurement of body image satisfaction.

### Strengths, Limitations and Future Research

A noteworthy strength of the present study was that consistent with recommendations for psychometric investigations [21,22], exploratory, and confirmatory factor analyses were conducted to thoroughly evaluate the factor structure of the BISQ and identify a model that best fits a multidimensional structure. Another strength of this study was the relatively large and heterogeneous sample size. However, the present study had important limitations as well. First, our participants were aged between 18 and 49 years old; thus, results obtained in this age group might not be generalized to a younger or older population. Future studies might be designed to support current results, adding evidence to the validation of the scale, considering discriminant validity, and convergent validity with other instruments assessing body image perceptions. In addition, considering possible gender difference and prevalence of BI satisfaction/dissatisfaction in the general population [28] as well as possible differences across Body Mass Index, we suggest conducting invariance analysis using the BISQ. Another limitation of this study was the cross-sectional nature of the data, which did not allow for examination of test–retest reliability. Future research to address these limitations would be valuable to better understand the performance of the BISQ in screening for body image satisfaction.

## 5. Conclusions

This study points out that there are several factors within the BISQ that are related to body satisfaction. Hence, this study can provide statistical support to the understanding of the different dimensions within the BISQ and contribute to further assessment of individual perception of body image satisfaction.

Overall, by relying on the factor structure of the 23-item BISQ, body image satisfaction dimensions were identified as distinct constructs representing different body parts; where individuals rated as highly satisfied or not. Additionally, the BISQ presented itself as a reliable 23-item measure on assessing how much individuals were satisfied with each body part. The results provide support for use of the BISQ, adding evidence of the factor structure of this instrument in the general domain

### Practical Implications

The BISQ measure could by useful to clinical practitioners, such as psychologists, by increasing the understanding of body image satisfaction in individuals with different ages, not only to determine the degree of overall body image satisfaction, but also to examine possible determinants of satisfaction or frustration with their appearance. The present research provided initial psychometric evidence supportive of a multidimensional measure of body image satisfaction, which can be used in both sexes. In addition, since the BISQ was validated in the general population, its items could be particularly useful as clinical tools for monitoring body image satisfaction over a course of intervention.

## Figures and Tables

**Table 1:** Psychometric proprieties of the tested models.

Model	*χ* ^2^	df	CFI	TLI	SRMR	RMSEA	CI90%
1. One factor EFA	3190.085	230	0.724	0.697	0.081	0.120	0.116, 0.123
2. Two factor EFA	2092.804	208	0.824	0.786	0.049	0.100	0.089, 0.104
3. Tree factor EFA	1375.675	187	0.889	0.850	0.039	0.084	0.080, 0.088
4. Four factor EFA	1014.771	167	0.921	0.880	0.030	0.075	0.071, 0.080
5. Five factor EFA	578.904	148	0.960	0.931	0.022	0.057	0.052, 0.062
7. Five-correlated factor CFA	1088.100 *	220	0.904	0.899	0.057	0.066	0.062, 0.070
8. Second-order factor CFA	1156.460 *	225	0.865	0.848	0.064	0.068	0.064, 0.072
9. Bifactor CFA	775.189 *	197	0.916	0.892	0.036	0.057	0.053, 0.061

**Notes:***χ*^2^ = qui-square test; df = degrees of freedom; CFI = Comparative Fit Index; TLI = Tucker Lewis Index; SRMR = Standardized Root Mean Square Residual; RMSEA = Root Mean Square Error of Approximation; CI90% = Confidence Interval at 90% of RMSEA; * *p* < 0.001.

**Table 2:** Descriptive statistics of each item and geomin loadings of the five-factor EFA model.

Item	*M*	*SD*	Range	S	K	F1	F2	F3	F4	F5
1—Hair	3.79	0.97	1–5	−0.86	0.42	0.47 *	0.14 *	0.09	0.16 *	−0.18 *
2—Teeth	3.44	1.06	1–5	−0.70	−0.23	0.45 *	0.04	−0.06	0.18 *	0.06
3—Eyes	4.05	0.81	1–5	−0.75	0.58	0.64 *	0.04	−0.06	0.05	0.02
4—Ears	3.91	0.84	1–5	−0.62	0.37	0.74 *	−0.04	0.01	−0.11 *	0.01
5—Nose	3.61	0.94	1–5	−0.68	0.36	0.61 *	−0.01	0.05	−0.03	0.08 *
6—Skin	3.72	0.92	1–5	−0.90	0.91	0.59 *	0.23 *	0.00	0.24 *	−0.05
7—Facial aspect	3.76	0.86	1–5	−0.87	0.97	0.48 *	0.02	0.01	0.32 *	−0.01
8—Arms	3.55	0.95	1–5	−0.44	−0.47	−0.08 *	0.74 *	0.00	0.08	0.09 *
9—Chest	3.39	1.03	1–5	−0.56	−0.32	0.08 *	0.52 *	0.06	0.14 *	−0.01
10—Shoulders	3.65	0.87	1–5	−0.55	0.10	0.04	0.79 *	0.03	−0.04	0.03
11—Abdominals	3.00	1.19	1–5	−0.22	−1.03	−0.08 *	0.01	0.62 *	0.33 *	−0.09
12—Hips	3.39	1.04	1–5	−0.51	−0.40	0.10 *	0.04	0.60 *	0.04	0.22 *
13—Waist	3.29	1.07	1–5	−0.43	−0.58	0.05	0.01	0.89 *	0.01	0.00
14—Glutes	3.38	1.06	1–5	−0.46	−0.45	0.00	0.15 *	0.39 *	−0.08 *	0.55 *
15—Thighs	3.41	1.09	1–5	−0.50	−0.56	0.06 *	−0.05 *	0.18 *	0.09	0.79 *
16—Legs	3.48	1.07	1–5	−0.59	−0.36	−0.05 *	0.09 *	−0.02	0.16 *	0.76 *
17—Body shape	3.45	1.04	1–5	−0.61	−0.33	0.06	0.06	0.49 *	0.14 *	0.10 *
18—Port	3.59	0.95	1–5	−0.71	0.28	0.09	0.13 *	0.20 *	0.48 *	0.04
19—Weight	3.25	1.17	1–5	−0.49	−0.75	−0.08	−0.13 *	0.38 *	0.52 *	0.12 *
20—Height	3.63	1.44	1–5	−0.17	−0.21	0.13 *	0.08	−0.01	0.46 *	−0.06
21—Physical fitness	3.48	1.05	1–5	−0.55	−0.41	0.09 *	0.07	−0.08	0.76 *	0.01
22—Vitality	3.75	0.95	1–5	−0.80	0.51	0.19 *	0.10 *	0.06	0.62 *	0.05
23—Overall appearance satisfaction	3.64	0.89	1–5	−0.87	0.88	0.19 *	0.10 *	0.06	0.59 *	0.05

**Note:***M* = Mean; *SD* = Standard Deviation; S = Skewness; K = Kurtosis; F = Factor; bold = highest loading; * *p* < 0.05.

**Table 3:** Factor indicators of the five-correlated factor and bifactor CFA model.

Factor	Five-Correlated Factor CFA			Bifactor CFA		
	λ	λ^2^	δ	GFλ	SFλ	δ
F1—“Face”	0.77					
Item 1—Hair	0.53 **	0.28	0.72	0.41 **	0.31 **	0.74
Item 2—Teeth	0.50 **	0.25	0.75	0.44 **	0.21 **	0.76
Item 3—Eyes	0.58 **	0.33	0.67	0.44 **	0.55 **	0.58
Item 4—Ears	0.53 **	0.29	0.71	0.43 **	0.68 **	0.49
Item 5—Nose	0.59 **	0.35	0.65	0.35 **	0.54 **	0.59
Item 6—Skin	0.61 **	0.37	0.63	0.52 **	0.46 **	0.66
Item 7—Facial aspect	0.69 **	0.48	0.52	0.59 **	0.33 **	0.54
F2—“Upper Torso”	0.80					
Item 8—Arms	0.77 **	0.60	0.40	0.68 **	0.68 **	0.44
Item 9—Chest	0.69 **	0.48	0.52	0.67 **	0.56 **	0.55
Item 10—Shoulders	0.79 **	0.62	0.39	0.64 **	0.67 **	0.26
F3—“Lower Torso”	0.88					
Item 11—Abdominals	0.77 **	0.59	0.26	0.63 **	0.45 **	0.40
Item 12—Hips	0.82 **	0.67	0.33	0.65 **	0.49 **	0.34
Item 13—Waist	0.86 **	0.74	0.14	0.64 **	0.65 **	0.17
Item 17—Body shape	0.77 **	0.59	0.52	0.71 **	0.50 **	0.40
F4—“Lower Body”	0.90					
Item 14—Glutes	0.82 **	0.68	0.14	0.59 **	0.56 **	0.34
Item 15—Thighs	0.93 **	0.86	0.29	0.60 **	0.73 **	0.10
Item 16—Legs	0.84 **	0.70	0.41	0.58 **	0.62 **	0.30
F5—“Body Appearance”	0.82					
Item 18—Port	0.69 **	0.48	0.47	0.67 **	0.53 **	0.53
Item 19—Weight	0.73 **	0.53	0.89	0.64 **	0.65 **	0.17
Item 20—Height	0.32 **	0.10	0.42	0.32 **	0.29*	0.89
Item 21—Physical fitness	0.76 **	0.58	0.63	0.72 **	0.61 **	0.43
Item 22—Vitality	0.61 **	0.37	0.38	0.62 **	0.50 **	0.61
Item 23—Overall appearance satisfaction	0.79 **	0.62	0.38	0.83 **	0.54 **	0.30

**Note:** SF = Specific Factor; GF = Global Factor; IECV β = Individual Explained Common Variance coefficients; λ = factor loadings; δ = uniqueness; target loadings are in bold; composite reliability coefficients in bold; ** *p* < 0.001.

**Table 4:** Descriptive statistics and correlations.

Factor	*M*	*SD*	S	K	1.	2.	3.	4.	5.
1. Face	3.75	0.59	−0.41	0.49	1				
2. Upper Torso	3.35	0.78	−0.411	0.03	0.67 **	1			
3. Lower Torso	3.28	0.92	−0.42	−0.39	0.56 **	0.67 **	1		
4. Lower Body	3.42	0.97	−0.47	−0.34	0.66 **	0.69 **	0.77 **	1	
5. Body Appearance	3.57	0.76	−0.35	10.07	0.44 **	0.58 **	0.76 **	0.72 **	1

**Notes:***M* = Mean; *SD* = Standard Deviation; S = Skewness; K = Kurtosis; ** *p* < 0.001.

## Data Availability

Due to issues of participant consent, data will not be shared publicly. Interested researchers may contact the last author associated in this study.
